# Adjoint Transformation Algorithm for Hand–Eye Calibration with Applications in Robotic Assisted Surgery

**DOI:** 10.1007/s10439-018-2097-4

**Published:** 2018-07-26

**Authors:** Krittin Pachtrachai, Francisco Vasconcelos, François Chadebecq, Max Allan, Stephen Hailes, Vijay Pawar, Danail Stoyanov

**Affiliations:** 1 Wellcome / EPSRC Centre for Interventional and Surgical Sciences (WEISS) and the Department of Computer Science, University College London, London, UK; 2 Intuitive Surgical, Sunnyvale, CA USA; 3 Department of Computer Science, University College London, London, UK

**Keywords:** Calibration problem, Hand–eye relationship, Stereoscopic camera, Surgical robot

## Abstract

Hand–eye calibration aims at determining the unknown rigid transformation between the coordinate systems of a robot arm and a camera. Existing hand–eye algorithms using closed-form solutions followed by iterative non-linear refinement provide accurate calibration results within a broad range of robotic applications. However, in the context of surgical robotics hand–eye calibration is still a challenging problem due to the required accuracy within the millimetre range, coupled with a large displacement between endoscopic cameras and the robot end-effector. This paper presents a new method for hand–eye calibration based on the adjoint transformation of twist motions that solves the problem iteratively through alternating estimations of rotation and translation. We show that this approach converges to a solution with a higher accuracy than closed form initializations within a broad range of synthetic and real experiments. We also propose a stereo hand–eye formulation that can be used in the context of both our proposed method and previous state-of-the-art closed form solutions. Experiments with real data are conducted with a stereo laparoscope, the KUKA robot arm manipulator, and the da Vinci surgical robot, showing that both our new alternating solution and the explicit representation of stereo camera hand–eye relations contribute to a higher calibration accuracy.

## Introduction

Surgical procedures are increasingly minimally invasive through the use of advanced instrumentation and imaging to operate inside the body.^23^ Robot-assisted minimally invasive surgery (RMIS) enables the tele-manipulation of surgical instruments with a high degree of dexterity, motion scaling and improved ergonomics compared to hand-held instrumentation.^19^ It facilitates to the reduction of post-operative recovery times and allows the execution of difficult micro-surgical tasks with enhanced stability and precision. Typically, RMIS is performed by articulated instruments and under direct observation using a stereo endoscopic camera. While the tele-manipulation robot can provide information about the position and movement of the distal end of the instrument tips, this is usually subject to calibration offsets due to the tendon based architectures used to provide minimally invasive access inside the anatomy. This information can be potentially recovered from the camera itself using vision but it still needs to be linked to the robot’s kinematic coordinates.^1^,^26^ Achieving this link requires accurate hand–eye calibration to find the rigid transformation between the stereo camera and the robot coordinate frames. While many algorithms exist for solving the hand–eye problem, improved accuracy is still a requirement that needs further attention.

There is an extensive literature on different hand–eye calibration methods. The most common procedure consists in acquiring a planar checker-board target under different orientations. The camera motion is determined from decomposing the homography that represents the mapping for the calibration plane between the two image, meanwhile the robot while the motion of the robot end-effector is obtained from the forward kinematics of the joint parameters. Given a set of camera motions, there are different alternatives to solve the hand–eye problem. One possible approach is to remove the unknown translation parameters from the hand–eye equations and estimate the rotation independently, while the translation is obtained in a posterior step through linear estimation.^3^,^4^,^16^,^24^,^31^,^35^,^36^ An alternative approach is to jointly estimate the rotation and translation components using a dual quaternion parametrisation,^5^,^7^ which generally yields more stable translation results. Both of these approaches can be posteriorly refined with iterative local optimization.^37^ The hand–eye problem can also be solved without any calibration target using a structure-from-motion (SfM) approach.^8^,^9^,^28^ However, this usually yields less accurate results since the 3D points in the scene are additional unknowns.

The hand–eye calibration methods mentioned above also do not account for different robot and camera motion uncertainties. Although these will generally depend on the particular hardware set-up, in surgical robotics the robot motions have consistently larger errors than the camera motions. This is due to the fact that nominal kinematic parameters of the da Vinci is perturbed by stress and strain in the cables which causes the inaccuracy in the whole kinematic chain.

Data selection also plays a vital role in the performance of hand–eye calibration.^27^ In order to obtain high accuracy, a wide range of rotational motions should be included into the calibration, whilst minimising distance between optical centre of the camera and calibration grid. Unfortunately, some of these criteria are sometimes not feasible in practice, since the movement of the end-effector in some robots is often confined within some particular volume. For example, the da Vinci surgical robot is constrained to move around a pre-determined remote centre of motion (RCM).

The use of stereo cameras in hand–eye calibration can greatly improve its accuracy, since each measurement establishes additional geometric constraints. Although most hand–eye calibration methods are formulated for monocular cameras, they can be directly applied to stereo vision^33^ by considering the two cameras as two independent motions from a monocular camera. This, however does not exploit the additional constraints of a calibrated stereo pair. The cost functions that explicitly models a stereo camera can be posteriorly refined along with camera parameters after an initial calibration with a monocular hand–eye algorithm.^15^,^17^ Alernatively, the problem can be formulated in a simpler form by obtaining only a pure rotation motions.^14^ This is not suitable for surgical robotics where the workspace is strictly limited and is often incompatible with significant pure rotation motions.

The use of screw motion is evident in visual servoing (vision-based control) where the robot changes pose according to the image obtained from a mounted camera.^34^ Such approach can be used to guide the robot to change pose with respect to one or more targeted objects without the use of any calibration. However, because of unknown calibration parameters (intrinsic, extrinsic and hand–eye), the algorithm can only guide the end-effector to be within a centimetre of a desired pose which is not sufficient for surgical purposes. Plus, with known calibration parameters, the link between robot and camera coordinate systems will be established and consequently, the information computed anywhere in the system can be linked to both robot frame and camera frame.

The algorithm developed in this paper is similar to the algorithms in our recently published works. The work^21^ makes use of tracking algorithm to detect the pose of a surgical tool and performs hand–eye calibration using a surgical tool as a calibration target and the algorithm developed later^22^ is focused on the data synchronisation before performing hand–eye calibration where the capture rate and activation time of the two sensors are different. Although the hand–eye calibration algorithm is discussed in the papers, they do not include stereo information in the formulation which can further improve the calibration performance.

This paper introduces the Adjoint Transformation Algorithm (ATA) using the hand–eye constraints in terms of the screw motion parameters, by defining a $6\times 6$ adjoint transformation that maps a screw motion from the robot end-effector to the camera coordinate frame.^30^

- The advantages of using the screw motion to define transformations is that it can handle parallel rotation axes and recover a partial solution by using the geometry of composite motion.^2^,^34^
- The advantage of decoupling the estimations mitigates the effect of noise from the translation component in the rotation estimation, unlike the commonly used dual quaternion method^5^ where the algorithm estimates the translation and rotation components simultaneously.
- Our formulation using adjoint transformation can avoid using the noisy motions which works well when the robot motions are less accurate than the camera motions or the other way around.
- Furthermore, this paper also introduces a formulation for the stereo hand–eye problem that is suitable for both initialisation and refinement, works with any of the state-of-the-art algorithms and does not require restricted motions.

The proposed solution is compared with other existing hand–eye calibration algorithms in the literature that are commonly used.^5^,^16^,^36^ The experiments are performed with both synthetic and real data. The real data is obtained with a stereo laparoscope attached to the flange of the KUKA LBR IIW A 7 R800, as well as with the da Vinci surgical robot. The code for the work and the comparison studies are packaged in a toolbox which is available online at https://github.com/surgical-vision/handeye-ata.

### Notation

Vectors are represented by a lower-case letter with an arrow, e.g. ${\vec {a}}$. Skew symmetric matrices of a vector ${\vec {a}}$ are represented by $[{\vec {a}}]_\times$. Matrices are represented by a bold capital letter, e.g. ${\mathbf {K}}$. Rotations are either represented as a $3\times 3$ orthonormal matrix, e. g. ${\mathbf {R}}$, or as a $3\times 1$ vector, e. g. ${\vec {r}}$, whose direction and norm are respectively the principal axis and the magnitude of the rotation. Bold lower-case letters represent quaternions, e. g. ${\mathbf {q}}$. Multiplication between two quaternions is written by using the . operator, e.g ${\mathbf {a}}.{\mathbf {b}}$. The inverse of a quaternion is represented as ${\mathbf {q}}^{-1}$. The rigid transformation between frame *i* and frame *j* is denoted by the $4\times 4$ matrix $^j{\mathbf {T}}_i$, i.e. calculating a point $p_i$ that is currently represented in the frame *i* in the frame *j* is simply $^j{\mathbf {T}}_i[p, 1]^T$. The transformation can also be represented in terms of a Lie algebra. The mapping between these two representations involves using the matrix exponential, i.e. ${{\mathrm{expm}}}{({\mathfrak {a}})} = {\mathbf {A}}$, where ${{\mathrm{expm}}}$ represents the matrix exponential function and ${\mathfrak {a}}$ represents the corresponding $4\times 4$ Lie algebra for a rigid transformation ${\mathbf {A}}$.

## Methods

**Figure 1:**
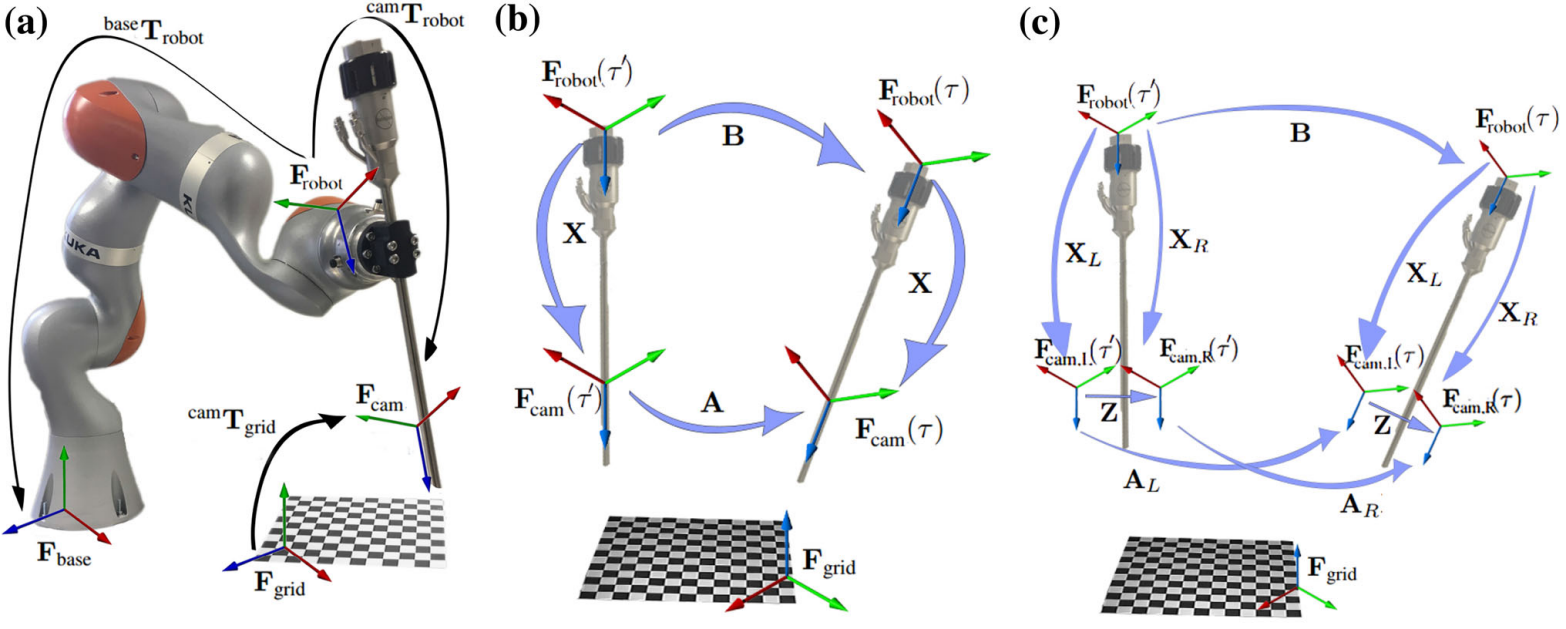
(a) Experimental set-up for the classic hand–eye problem. The camera is attached to the end-effector of the robot, in this case, the KUKA’s flange. Hand–eye calibration is the method used determine the missing transformation $^{\text {cam}}{\mathbf {T}}_{\text {robot}}$ which is defined as a pose of robot’s frame with respect to the camera’s pose. (b) The schematic showing the example of relative transformations for the robot frame and camera frame as mathematically represented by Eqs. (3) and (4). (c) The schematic for stereoscopic hand–eye formulation as mathematically represented by Eqs. (19) and (24). ${\mathbf {A}}_{LR}$ is the transformation between ${\mathbf {F}}_{\text {cam, L}}(\tau )$ and ${\mathbf {F}}_{\text {cam, R}}(\tau ^{\prime })$, while ${\mathbf {A}}_{RL}$ is the transformation between ${\mathbf {F}}_{\text {cam, R}}(\tau )$ and ${\mathbf {F}}_{\text {cam, L}}(\tau ^{\prime })$.

As seen in Fig. 1a, the hand–eye problem aims at estimating the unknown rigid transformation $^{\text {cam}}{\mathbf {T}}_{\text {robot}}$ between the camera centre of projection and the robot end-effector in eye-to-hand configuration, i.e. the camera is rigidly mounted in a fixed reference position. The transformations $^{\text {cam}}{\mathbf {T}}_{\text {grid}}$ and $^{\text {base}}{\mathbf {T}}_{\text {robot}}$ are determined from camera calibration and robot forward kinematics respectively. Camera calibration estimates both the camera intrinsic parameters and the relative pose $^{\text {cam}}{\mathbf {T}}_{\text {grid}}$ between the optical centre and the grid coordinate system.^25^ Forward kinematics are used to determine the pose $^{\text {base}}{\mathbf {T}}_{\text {robot}}$ of the end-effector with respect to the robot base frame.^32^ For *n* joints manipulator, the pose of end-effector is formulated as:

1 $$\begin{aligned} ^0{\mathbf{{T}}_n}{ = ^0}{\mathbf{{T}}_1}{\,^1}{\mathbf{{T}}_2}{ \ldots ^{n - 1}}{\mathbf{{T}}_n} \end{aligned}$$

where $^j{\mathbf {T}}_i$ represents a transformation for the *i*th link. The hand–eye problem is conventionally defined as the following equation^31^:

2 $$\begin{aligned} {\mathbf {AX}} = {\mathbf {XB}} \end{aligned}$$

with ${\mathbf {X}} = ^{\text {cam}}{\mathbf {T}}_{\text {robot}}$. ${\mathbf {A}}$ and ${\mathbf {B}}$ are $4\times 4$ rigid transformations representing camera and robot motion respectively, between two different calibration acquisitions $\tau$ and $\tau ^\prime$ (Fig. 1b), such that

3 $$\begin{aligned} {\mathbf {A}}&= \ \,\,\,\, ^{\text {cam}}{\mathbf {T}}_{\text {grid}}(\tau )(^{\text {cam}}{\mathbf {T}}_{\text {grid}}(\tau ^\prime ))^{-1}\end{aligned}$$

4 $$\begin{aligned} {\mathbf {B}}&=\,^{\text {robot}}{\mathbf {T}}_{\text {base}}(\tau )(^{\text {robot}}{\mathbf {T}}_{\text {base}}(\tau ^\prime ))^{-1} \end{aligned}$$

At least two motions whose rotation axes are neither parallel nor anti-parallel are required to solve this problem.^35^ With *N* robot-camera measurements under different poses, Eqs. (3) and (4) can be established for all different pairwise combinations of the *N* measurements ${N \atopwithdelims ()2}$.

### Problems in Hand–Eye Calibration

Without a noise in the system, Eq. (2) can be solved algebraically to recover the hand–eye transformation using a classic hand–eye solver.^35^ However, the equation is rather sensitive to noise such that a small noise in both robot motion and camera motion can invalidate the equation and make solving hand–eye problem more challenging in practice.

**Figure 2:**
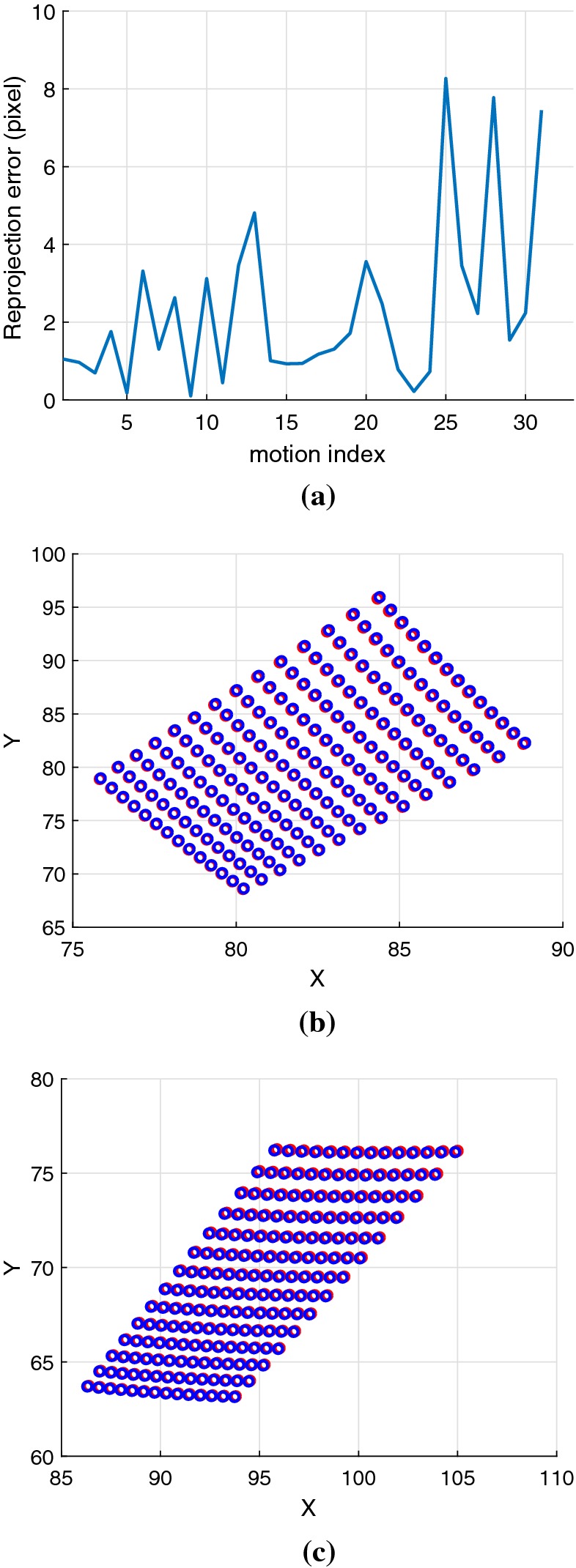
(a) Reprojection error when adding a small Gaussian noise of the standard deviation 0.025 mm into the system. (b) and (c) Examples of how the grids are projected back to the images after adding noise; Red dots represent ground truth and blue dots represent the re-projected grid after adding noise.

The noise in camera motion mostly occurs because of the error in the extrinsic parameters estimation, Although they are often considered negligible as the intrinsic calibration can refine up to sub-pixel accuracy. On the other hand, due to geometric errors (imprecision in manufacturing) and non-geometric errors (backlash, elasticity, joint compliance), the transformation $^{\text {base}}{\mathbf {T}}_{\text {robot}}$ is not perfectly accurate.^12^ With synthetic data (Fig. 2a) we show that even a sub-millimetre error in robot translation has a noticeable impact on the calibration results. In practice this effect can be minimized with a more accurate robot calibration.^10^

**Figure 3:**
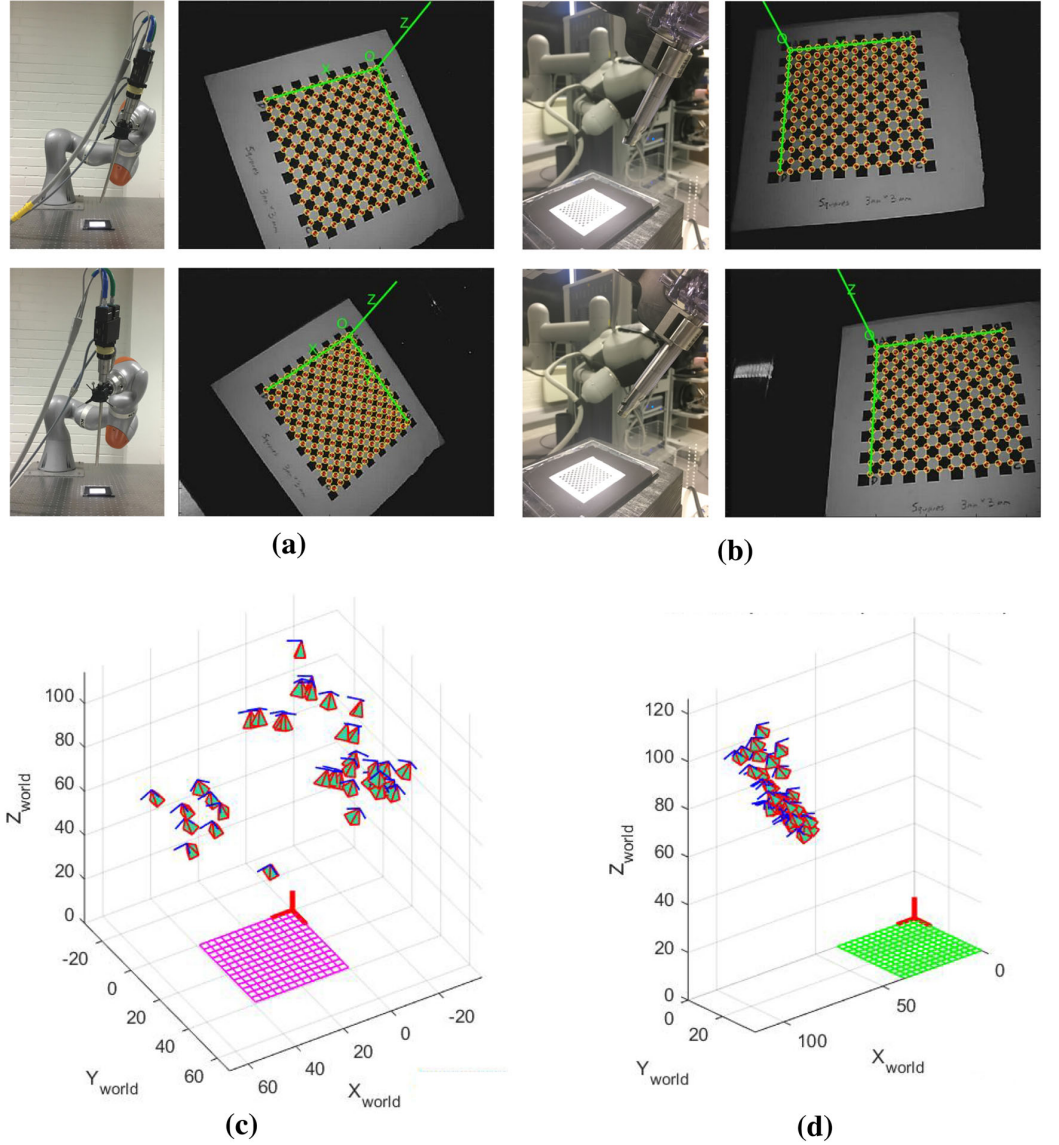
(a) Example images of the KUKA’s pose and its corresponding image side by side. KUKA is moved to several positions to collect images of the grid in several points of view, while the pose of the KUKA is acquired using its API. (b) Example images of da vinci’s pose and its corresponding image side by side. (c) Camera poses with respect to the calibration grid obtained with the KUKA robot. Images can be acquired for a wide range of camera motions, resulting in hand–eye calibrations with higher accuracy. (d) Camera poses with respect to the calibration grid obtained with the da Vinci robot. Camera motion is constrained to a smaller range of rotations and translations and thus hand-eyed calibration is more challenging.

Apart from the issue of noise in the system, the range of motion is also a challenging problem in hand–eye calibration as well. It is proved in the literature that the wider range of motion increases the calibration accuracy,^36^ but this is not feasible in all of the robot set-up. For example, the camera motion captured from robot arm KUKA LBR IIWA R800 is much wider than the one from the da Vinci surgical robot as shown in Figs. 3a–3d. The effect of range of motion is more evident in the results section.

### Hand–Eye Calibration with an Adjoint Transformation

The algorithm jointly applies the equation for solving the rotation part^16^ and the equation from an adjoint transformation to create a more robust constraint for hand–eye estimation.Similarly to most state-of-the-art approaches, Levenberg–Marquadt is also applied at the end of the algorithm to minimise the calibration error.^6^,^18^

This method is similar to the camera calibration algorithm which iteratively solves for extrinsic and intrinsic parameters in alternate steps, followed by a local optimisation refinement step.^25^

Equation (2) can be decomposed into two parts as follows:

5 $$\begin{aligned} {\mathbf {R}}_A{\mathbf {R}}_X = {\mathbf {R}}_X{\mathbf {R}}_B \end{aligned}$$

6 $$\begin{aligned} {\mathbf {R}}_A{\vec {t}}_X + {\vec {t}}_A = {\mathbf {R}}_X{\vec {t}}_B + {\vec {t}}_X \end{aligned}$$

where ${\mathbf {R}}_X$ and ${\vec {t}}_X$ are the rotation component and the translation component of the hand–eye transformation, respectively. Equation (5) can be represented in a quaternion form. Let ${\mathbf {a}}$, ${\mathbf {x}}$ and ${\mathbf {b}}$ be quaternions that represent the rotation components of the transformations ${\mathbf {A}},{\mathbf {X}}$ and ${\mathbf {B}}$. Thus, we have the following equation:

7 $$\begin{aligned} {\mathbf {a}}.{\mathbf {x}} = {\mathbf {x}}.{\mathbf {b}} \end{aligned}$$

Equation (7) can be rearranged into a matrix form:

8 $$\begin{aligned} \begin{bmatrix} a_0 - b_0&\quad -({\vec {a}} - {\vec {b}})^T \\ {\vec {a}} - {\vec {b}}&\quad [{\vec {a}} + {\vec {b}}]_\times + (a_0 - b_0){\mathbf {I}}_3 \end{bmatrix} {\mathbf {x}}&= {\vec {0}} \end{aligned}$$

where ${\mathbf {I}}_3$ is the identity matrix of size $3\times 3$, $a_0, b_0$ and ${\vec {a}}, {\vec {b}}$ are scalar and vector components of the quaternions ${\mathbf {a}},{\mathbf {b}}$, respectively. ${\mathbf {K}}({\mathbf {a}}, {\mathbf {b}})$ is a $4N\times 4$ matrix formed by using *N* motions.

9 $$\begin{aligned} {\mathbf {K}}({\mathbf {a}},{\mathbf {b}}){\mathbf {x}} = {\vec {0}} \end{aligned}$$

The classical solution for solving the rotation with Eq. (9) relies on SVD decomposition.^16^ In order to find the translation component, we introduce additional constraints by formulating the hand–eye equations in terms of an adjoint transformation. Post-multiplying Eq. (2) by ${\mathbf {X}}^{-1}$ yields ${\mathbf {A}} = {\mathbf {XBX}}^{-1}$. Let ${\mathfrak {a}}$ and ${\mathfrak {b}}$ be corresponding Lie algebras for rigid transformations ${\mathbf {A}}$ and ${\mathbf {B}}$, respectively, then by using the relationship between these two matrices provided in the Appendix section we can convert the hand–eye equation into:

10 $$\begin{aligned} {{\mathrm{expm}}}{({\mathfrak {a}})} = {\mathbf {X}}{{\mathrm{expm}}}{(\mathfrak {b})}{\mathbf {X}}^{-1} \end{aligned}$$

Because a rigid transformation is always invertible, ${\mathbf {X}}{{\mathrm{expm}}}{(\mathfrak {b})}{\mathbf {X}}^{-1} = {{\mathrm{expm}}}{({\mathbf {X}}{\mathfrak {b}}{\mathbf {X}}^{-1})}$.^30^ From here, we cannot safely assume that ${\mathfrak {a}} = {\mathbf {X}}\mathfrak {b}{\mathbf {X}}^{-1}$ since the exponential mapping is surjective, i.e. there can be more than one Lie algebra matrix that maps to the rigid transformation ${{\mathrm{expm}}}{({\mathfrak {a}})}$. Therefore, we have to prove that the mapping in our application is uniquely defined for every possible transformation. As rigid transformations consist of 3-DOF in rotation and 3-DOF in translation, and according to Eqs. (33–36) in the Appendix, the mapping between rotation components is independent from the mapping of translation components,^30^ therefore we can prove the uniqueness of the rotation mapping separately.

The exponent term of the rotation matrix is uniquely defined when the trace of the rotation matrix is not equal to $-1$, i.e. when the angle of rotation is not $\pm \pi$.^24^ Therefore, if we avoid $180^\circ$ of rotations during the calibration procedure, we can safely assume the uniqueness of this mapping. Note that these particular rotations are already extremely difficult to appear in practice, since the robot motions are constrained by the camera field of view relative to the calibration target. Hence,

11 $$\begin{aligned} {\mathfrak {a}} = {\mathbf {X}}{\mathfrak {b}}{\mathbf {X}}^{-1} \end{aligned}$$

12 $$\begin{aligned} \begin{bmatrix} [{\vec {\omega }}_A]_{\times }&\quad {\vec {v}}_A \\ {\vec {0}}^T&\quad 0 \end{bmatrix} = \begin{bmatrix} {\mathbf {R}}_X&\quad {\vec {t}}_X \\ {\vec {0}}^T&\quad 1 \end{bmatrix} \begin{bmatrix} [{\vec {\omega }}_B]_{\times }&\quad {\vec {v}}_B \\ {\vec {0}}^T&\quad 0 \end{bmatrix} \begin{bmatrix} {\mathbf {R}}_X^T&\quad -{\mathbf {R}}_X^T{\vec {t}}_X \\ {\vec {0}}^T&\quad 1 \end{bmatrix} \end{aligned}$$

13 $$\begin{aligned} \begin{bmatrix} {\vec {\omega }}_{A} \\ {\vec {v}}_{A} \end{bmatrix} = \begin{bmatrix} {\mathbf {R}}_X&\quad {\mathbf {0}}_{3\times 3} \\ [{\vec {t}}_X]_{\times }{\mathbf {R}}_X&\quad {\mathbf {R}}_X \end{bmatrix} \begin{bmatrix} {\vec {\omega }}_{B} \\ {\vec {v}}_{B} \end{bmatrix} \end{aligned}$$

where ${\vec {\omega }}_A, {\vec {\omega }}_B, {\vec {v}}_A$ and ${\vec {v}}_B$ are the Lie Algebra components of the transformations. The relation between ${\vec {\omega }}_A$ and ${\vec {\omega }}_B$ in the first three rows of Eq. (13) is known as the orthogonal Procrustes Problem^29^ and it has already been satisfied when solving for the rotation with Eq. (9). The estimation of the translation component in ATA is achieved using the relation between ${\vec {v}}_A$ and ${\vec {v}}_B$ (the last three rows of Eq. (13))

14 $$\begin{aligned} {\vec {v}}_A = [{\vec {t}}_X]_{\times }{\mathbf {R}}_X{\vec {\omega }}_B + {\mathbf {R}}_X{\vec {v}}_B \end{aligned}$$

According to their respective documentations, the KUKA has a repeatability of 0.1 mm and the da Vinci positioning system is accurate within only a 5 cm cube while the laparoscopic camera calibration can be refined up to sub-pixel accuracy.^11^,^13^ This error in the positioning system creates more than sub-pixel errors when the grid is projected onto images as shown in Fig. 2a. Therefore, the relative camera motion ${\mathbf {A}}$ is more accurate than the robot motion ${\mathbf {B}}$. Hence, from Eq. (14), we can avoid using the rotation part of ${\mathbf {B}}$ by substituting ${\mathbf {R}}_X{\vec {\omega }}_B$ with ${\vec {\omega }}_A$.

15 $$\begin{aligned} {\vec {v}}_A = [{\vec {t}}_X]_{\times }{\vec {\omega }}_A + {\mathbf {R}}_X{\vec {v}}_B \end{aligned}$$

To simultaneously solve Eq. (15) with Eq. (9), we need to transform Eq. (15) into quaternion form:

16 $$\begin{aligned} \begin{bmatrix} 0&-({\vec {v}}_A - [{\vec {t}}_X]{\vec {\omega }}_A - {\vec {v}}_B)^T \\ {\vec {v}}_A - [{\vec {t}}_X]{\vec {\omega }}_A - {\vec {v}}_B&[{\vec {v}}_A - [{\vec {t}}_X]{\vec {\omega }}_A + {\vec {v}}_B]_{\times } \end{bmatrix} {\mathbf {x}}&= {\vec {0}} \end{aligned}$$

Therefore, the rotation component is refined using Eqs. (9) and (16) simultaneously, and the next equation is for estimating the translation component. Since $[{\vec {t}}]_{\times }{\vec {\omega }}_A$ is equivalent to $-[{\vec {\omega }}_A]_{\times }{\vec {t}}$, we can arrive at the equation:

17 $$\begin{aligned}{}[{\vec {\omega }}_A]_{\times }{\vec {t}}_X = {\mathbf {R}}_X{\vec {v}}_B - {\vec {v}}_A \end{aligned}$$

**Figure 4:**
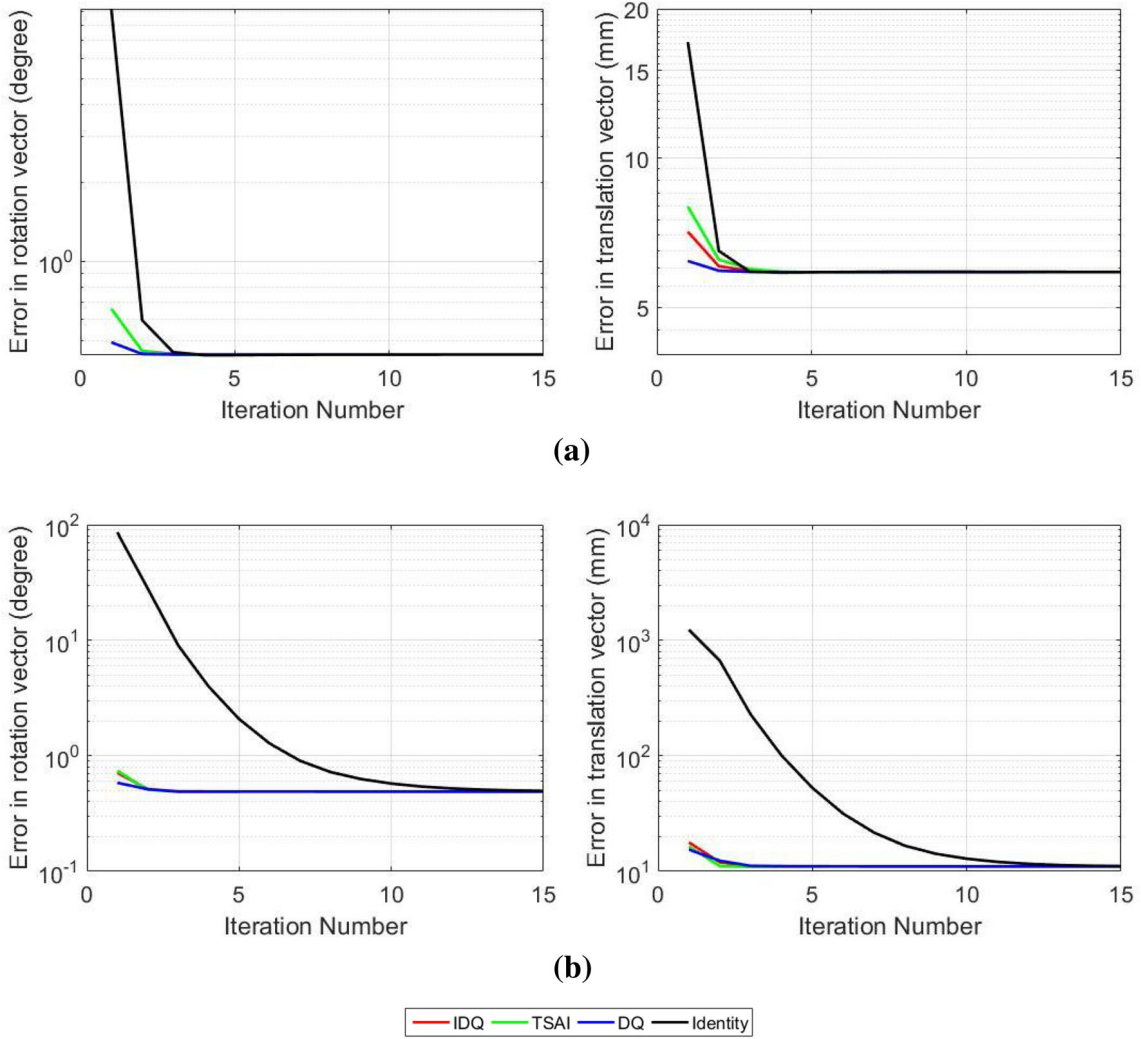
Comparison of convergence rates of ATA with different initialisation methods is displayed using mean of the translation and rotation errors for improved dual quaternions (IDQ), Tsai’s linear method (TSAI) and dual quaternions (DQ). (a) The ground truth solution is close to identity matrix. (b) The ground truth solution is far from the identity matrix.

Equation (17) indicates that the translation part of the hand–eye relationship must satisfy this system of equations. By collecting *N* motions, we have $3\times N$ different equations. Therefore, the solution for the translation part will satisfy Eq. (17) and at the same time enhance the accuracy in the rotation component. Then, the algorithm will go back to solve for the rotation part using the refined version of the translation and continue this process until the solution converges. The convergence criteria of the algorithm is when there is less than $10^{-4}$ change in both rotation and translation components for more than 20 iterations, although as with all iterative methods this can be adjusted. According to the results displayed in Fig. 4, the solution of ATA converges to the same result regardless of hand–eye initialisation, which is an important and practically valuable property of our method.

Finally, the hand–eye transformation can be refined further by using the Levenberg–Marquadt algorithm to minimise the error in hand–eye equation. The algorithm finds the Lie algebra ${\mathfrak {x}}$ which has the corresponding rigid transformation ${\mathbf {X}}$ that optimises the hand–eye equation. For all possible motions *N*, our objective function $\Phi ({\mathfrak {x}})$ can be written in Eq. (18).

18 $$\begin{aligned} \Phi ({\mathfrak {x}}) = \sum _{N} ||({\mathbf {AX}})^{-1}{\mathbf {XB}} - \mathbf {I}_4||^2 \end{aligned}$$

To summarise, ATA initialisation using monocular vision is described in Algorithm 1. In the next Section, we extend this formulation to stereo cameras.

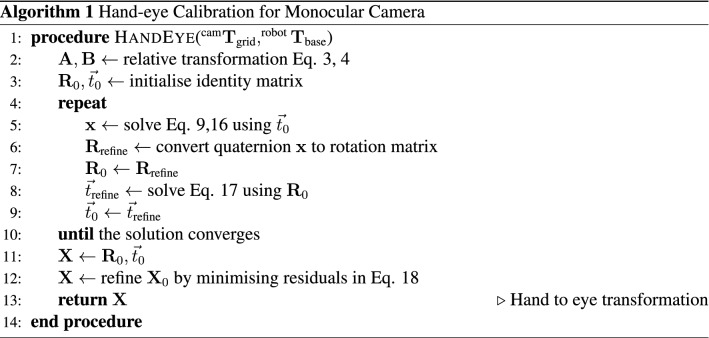



### Stereoscopic Formulation of Hand–Eye Calibration

The conventional hand–eye constraint from Eq. (2) can be extended to stereo vision, which enhances the calibration accuracy by introducing additional relative motion constraints. Since we have two cameras and they are rigidly held at the robot’s end-effector, each robot motion ${\mathbf {B}}$ can create two sets of camera motions in two different frames such that Eq. (2) can be formed for left and right cameras and represented as following,

19 $$\begin{aligned} {\mathbf {A}}_L{\mathbf {X}}_L = {\mathbf {X}}_L{\mathbf {B}} \end{aligned}$$

20 $$\begin{aligned} {\mathbf {A}}_R{\mathbf {X}}_R = {\mathbf {X}}_R{\mathbf {B}} \end{aligned}$$

**Figure 5:**
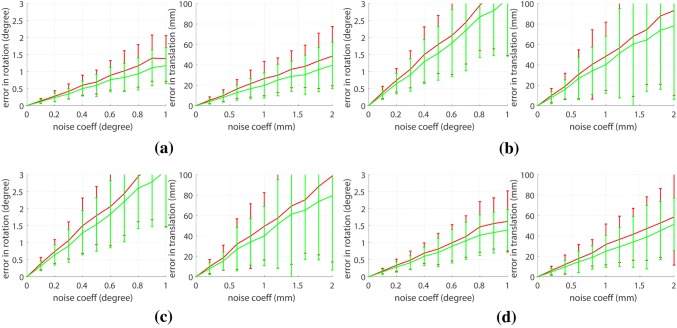
Comparison of monocular and stereoscopic camera formulation using different hand–eye calibration algorithms. Distribution (mean, standard deviation) of the translation and rotation errors are shown in red for the monocular case and in green for the stereoscopic case. ANOVA is applied when the noise coefficient in rotation is 0.2$^\circ$ and 0.4 mm in translation. (a) ATA ($p = 3.99\times 10^{-5}$ for rotation, $p = 5.43\times 10^{-5}$ for translation), (b) improved dual quaternion ($p = 4.83\times 10^{-2}$ for rotation, $p = 4.01\times 10^{-3}$ for translation), (c) Tsai ($p = 4.85\times 10^{-2}$ for rotation, $p = 1.04\times 10^{-2}$ for translation) and (d) dual quaternion ($p = 3.32\times 10^{-4}$ for rotation, $p = 1.54\times 10^{-4}$ for translation).

With a pre-calibrated stereo camera, the rigid transformation between the two cameras is already determined. Hence, the relationship between solutions in Eqs. (19) and (20) can be written as following,

21 $$\begin{aligned} {\mathbf {Z}}{\mathbf {X}}_L = {\mathbf {X}}_R \end{aligned}$$

where ${\mathbf {Z}}$ represents a rigid transformation between the left and right camera. Therefore, we can make use of Eq. (21) to the other three camera motions which relate to the same hand–eye solution as shown in Eqs. (22)–(24).

22 $$\begin{aligned} {\mathbf {Z}}^{-1}{\mathbf {A}}_R{\mathbf {Z}}{\mathbf {X}}_L&= {\mathbf {X}}_L{\mathbf {B}}\end{aligned}$$

23 $$\begin{aligned} {\mathbf {A}}_{LR}\mathbf {Z}{\mathbf {X}}_L&= {\mathbf {X}}_L{\mathbf {B}}\end{aligned}$$

24 $$\begin{aligned} {\mathbf {Z}}^{-1}{\mathbf {A}}_{RL}{\mathbf {X}}_L&= {\mathbf {X}}_L{\mathbf {B}} \end{aligned}$$

where ${\mathbf {A}}_{LR} = ^{\text {cam,L}}{\mathbf {T}}_{\text {grid}}(\tau )(^{\text {cam,R}}{\mathbf {T}}_{\text {grid}}(\tau ^\prime ))^{-1}$ and ${\mathbf {A}}_{RL} = ^{\text {cam,R}}{\mathbf {T}}_{\text {grid}}(\tau )(^{\text {cam,L}}{\mathbf {T}}_{\text {grid}}(\tau ^\prime ))^{-1}$. The schematic of these relationships is shown in Fig. 1c. By simultaneously solving Eqs. (19) and (22)–(24), it is shown in Fig. 5 that the calibration results improve regardless of the selection of algorithms.

As in the monocular vision case, the initial solution can be refined with Levenberg–Marquardt by minimising the error in the hand–eye equation as shown in Eq. (18) except that we have the additional motions from stereo which remains fixed. To summarise, ATA initialisation using stereo camera is described in Algorithm 2.

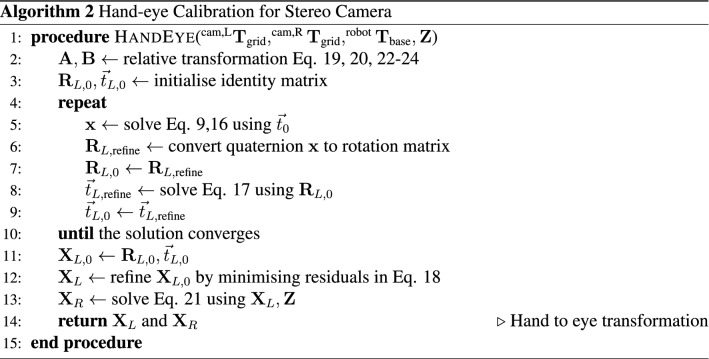



### Experimental Procedures

To generate the synthetic data for the experiments, we generate the two constant transformations $^{\text {base}}{\mathbf {T}}_{\text {grid}}$ and the ground truth for the hand–eye transformation $^{\text {cam}}{\mathbf {T}}_{\text {robot}}$ along with random robot poses $^{\text {base}}{\mathbf {T}}_{\text {robot}}$. The criteria for generating data depends on the simulated parameters in each experiment. The generated data is in the form of $6\times N$ matrix where *N* is the number of data used in the calibration. The first three rows stand for the translation in X, Y and Z and the last three rows is the Rodrigues’ representation of the rotation matrix. Once we get the transformations $^{\text {base}}{\mathbf {T}}_{\text {robot}}$, we then proceed to calculate $^{\text {cam}}{\mathbf {T}}_{\text {grid}}$ by multiplying all these three transformation together as shown below.

25 $$\begin{aligned} ^{\text {cam}}{\mathbf {T}}_{\text {grid}} = ^{\text {cam}}{\mathbf {T}}_{\text {robot}}(^{\text {base}}{\mathbf {T}}_{\text {robot}})^{-1}\ ^{\text {base}}{\mathbf {T}}_{\text {grid}} \end{aligned}$$

The transformations are corrupted by Gaussian noise before they are fed into the hand–eye calibration function. Equation (26) shows how the noise is added to an arbitrary transformation ${\mathbf {T}}$,

26 $$\begin{aligned} {\mathbf {T}}_{\text {corrupted}} = {\mathbf {T}} \begin{bmatrix} \texttt {rodrigues}(\sigma _r v_r)&\quad \sigma _t v_t \\ {\vec {0}}_{{1\times 3}}&\quad 1 \end{bmatrix} \end{aligned}$$

where $\sigma _r, \sigma _t$ are standard deviation of a zero-mean Gaussian noise for rotation and translation and $v_r, v_t$ are $3\times 1$ rotation vector and translation vector that are generated randomly. The tested algorithm then estimates $^{\text {cam}}{\mathbf {T}}_{\text {robot}}$ and we compare the estimated transformation with the ground truth. This process is run for 100 times for each simulation parameter.

The translation error is computed as the norm of the difference between ground truth and the estimated translation. Rotation error is computed as the magnitude of the residual rotation between ground truth and the estimation using Rodrigues’ formula.^20^ The equations for computing error in translation and rotation are shown in Eqs. (27)–(29), respectively.

27 $$\begin{aligned} E_{\text {translation}}&= ||t_1 - t_2|| \end{aligned}$$

28 $$\begin{aligned} {\vec {\delta \omega }}&=\text {rodrigues}({\mathbf {R}}_1{\mathbf {R}}_2^{-1})\end{aligned}$$

29 $$\begin{aligned} E_{\text {rotation}}&=||{\vec {\delta \omega }}|| \end{aligned}$$

where $t_1, t_2$ are $3\times 1$ vectors representing the translation components, ${\mathbf {R}}_1, {\mathbf {R}}_2$ are the rotation matrices of each transformation and rodrigues is a function that returns a $3\times 1$ vector describing the rotation error.

The experiments are run on a 2.6 GHz Intel Core i7-4510U laptop and ATA takes the processor around one second to complete the initialisation for refinement whereas the others take less than one second to converge. The number of motions does not increase computational cost but noisy data increases the time of convergence of both ATA alternation and Levenberg–Marquardt refinement. However, the computational time is not a priority of the work as the hand–eye calibration is typically an offline procedure.

ATA is also tested with real robots to study its robustness and accuracy in the presence of noise sources that might not be Gaussian. The robots used in the experiments are the KUKA LBR IIWA 7 R800 and the da Vinci Surgical Robot Standard as shown in Figs. 3a and 3b, respectively. A zero degree endoscope is attached to the end-effector of both robots.

Unlike in the experiment with synthetic data, the accurate ground truth for hand–eye transformation is not known. Therefore, we assess the performance of algorithms by predicting the camera pose from robot-pose as shown in Eq. (30).^5^ For both robots, we collect 40 measurements and randomly select a smaller number *N* of measurements as input to each calibration method in a succession of calibration trials (*N* is run from 3 to 13, i.e. 2 to 12 motions for successive motions). The selection of measurements is randomly repeated for 100 times to get meaningful results. After calibration, the solution is then used to predict the camera pose of unused measurements (validation set) which is compared with the pose retrieved from extrinsic parameters estimation to get the rotation and translation errors. As the camera pose prediction is similar to the estimation of the extrinsic parameters, we can assess the performance of the calibration algorithm in terms of re-projection errors as well. The evaluation of each sample is then averaged across the number of samples.

30 $$\begin{aligned} ^{\text {cam}}{\mathbf {T}}_{\text {grid}}(\tau _\text {predicted}) = (\mathbf {XB}{\mathbf {X}}^{-1}) ^{\text {cam}}{\mathbf {T}}_{\text {grid}}(\tau ^\prime ) \end{aligned}$$

For each experiment, we also run the one-way analysis of variance (ANOVA) on the selected raw calibration results to test whether the difference in the comparisons is statistically significant, i.e. the analysis is applied to $100\times 4$ raw calibration errors in translation and rotation components for each experiment since we aim to compare the calibration performance from 4 algorithms in 100 samples. The selection criteria for the analysis is based on the capability of the presently available robotic systems. We only select a case for each experiment that features the noise level that is close to the noise resilience of the robot and the configuration the robot can achieve in order to get an optimal calibration result.

## Results

### Experiment with Synthetic Data

This section compares synthetic calibration results between ATA and the algorithms in the literature. We start by displaying the effect of the error in the robotic positioning system and the convergence rate when ATA is initialised with different hand–eye methods or the identity matrix. Later we compare calibration accuracy of the existing methods with both monocular and stereo information.^5^,^16^,^36^ Finally, we compare all algorithms with the stereo set-up. In all experimental sections, we denote our algorithm as “ATA” and as following “TSAI”,^36^ “IDQ”^16^ and “DQ”.^5^ Since Fig. 5 shows that the use of stereo information increases the calibration accuracy, the results shown in subsequent Figures are generated from the use of stereoscopic formulation only. The result of ANOVA on selected simulated parameter is reported in the caption of each plot.

Giving that the *p* value for each test is less than 5%, we can deduce that the difference in calibration performance from each algorithm is statistically significant. The same technique is applied in the experiment with real data as well.

**Figure 6:**
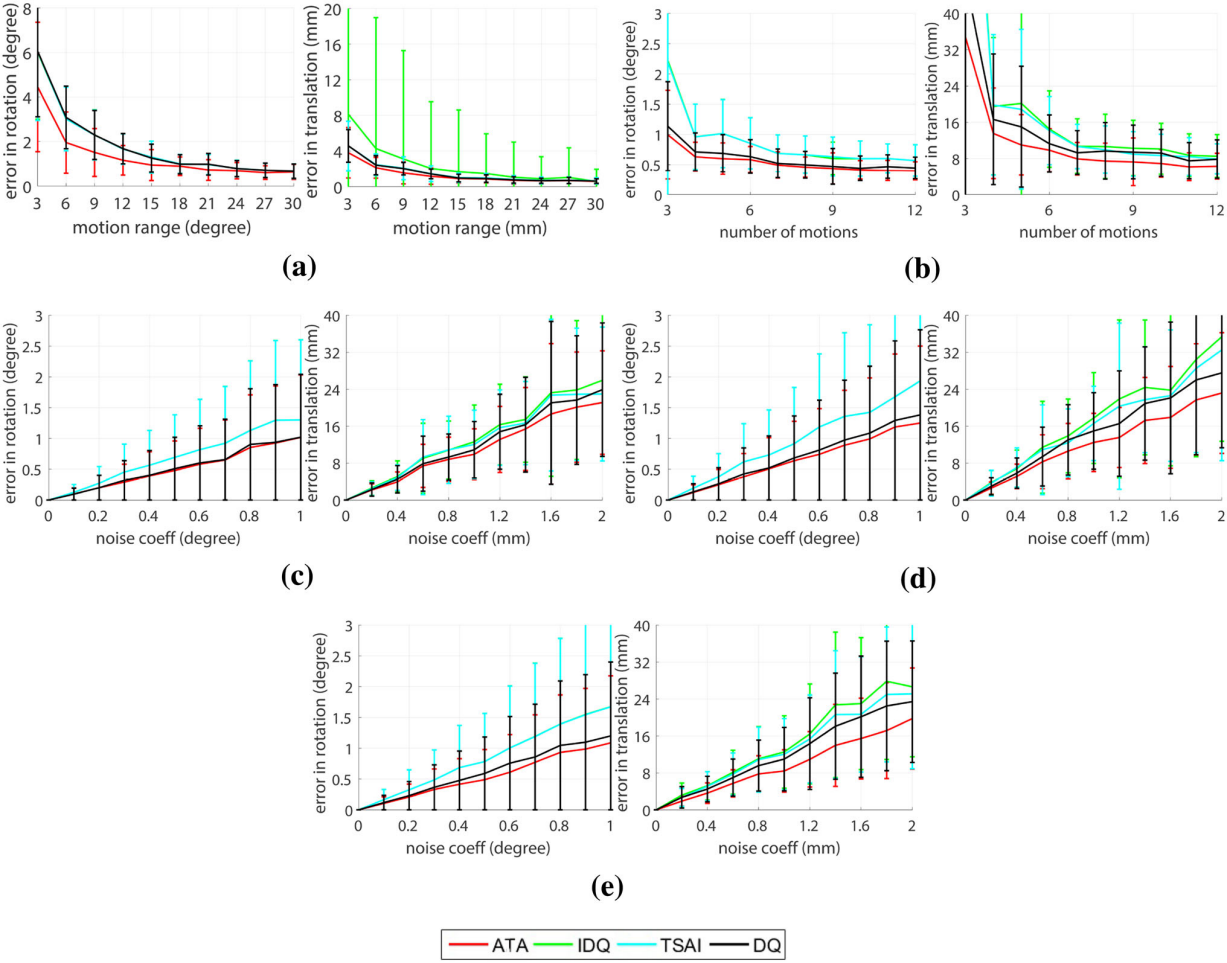
Stereo hand–eye performance comparison with synthetic data. Distribution (mean, standard deviation) of translation and rotation errors for ATA, improved dual quaternions (IDQ), Tsai’s linear method (TSAI) and dual quaternions (DQ): (a) Increasing motion range (at the motion range of 30$^\circ$ and 30 mm, $p = 3.55\times 10^{-16}$ for rotation and $p = 1.12\times 10^{-18}$ for translation). (b) Increasing number of motions (at the number of motions of 12, $p = 1.51\times 10^{-17}$ for rotation and $p = 5.19\times 10^{-7}$ for translation). (c) Increasing noise in robot motion (at the noise coefficients of 0.2$^\circ$ and 0.4 mm, $p = 1.91\times 10^{-7}$ for rotation and $p = 2.02\times 10^{-5}$ for translation). (d) Increasing noise in both robot and camera motion (at the noise coefficients of 0.2$^\circ$ and 0.4 mm, $p = 1.46\times 10^{-8}$ for rotation and $p = 1.57\times 10^{-6}$ for translation). (e) Increasing noise in robot motion, camera motion and stereo calibration (at the noise coefficients of 0.2$^\circ$ and 0.4 mm, $p = 6.33\times 10^{-10}$ for rotation and $p =3.19\times 10^{-4}$ for translation).

#### Effect of Error in the Robotic Positioning System

To compare the effect of the inaccuracy in robot parameters, poses of a robot are generated and corrupted by 0.025 mm Gaussian noise to simulate the robot with 0.1 mm repeatability in the positioning system. After that, we compare the projected checkerboard from using uncorrupted and corrupted poses. Figure 2a shows that most of the re-projection error are higher than 1 pixel which shows that the noise in the robotic positioning system is higher than the noise in the camera calibration.

#### Convergence Rate with Different Initialisation Methods

As ATA requires an initialisation to start the iterative solver, we also conduct an experiment to show how the error converges when different methods are applied to compute the initial solution. We use the outputs of the existing hand–eye methods as well as the identity matrix to initialise the solution. We test the algorithm in two situations: when the hand–eye matrix is close to identity (Fig. 4a) and when it is far from identity (Fig. 4b). The results show that the solution of our algorithm converges regardless of the starting point.

#### Inclusion of Stereo Information

Figure 5 shows the comparison between hand–eye calibration using monocular and stereoscopic formulation for increasing additive noise. They show that with respect to the same experimental conditions, using stereo vision provides more accurate results. According to Eqs. (22)–(24), one motion of stereo vision can provide three additional motion constraints to the hand–eye calibration and it is shown in Fig. 5b that increasing number of motions increases the calibration accuracy. Moreover, the figure also shows that ATA is more robust than the other methods when the noise coefficient is increased. This result is more evident in Figs. 6c, 6d, and 6e.

#### Increasing Motion Range

It is noted in the literature regarding hand–eye calibration that a wider motion range will enhance the robustness of the solver. In data selection for the hand–eye problem, we should select pairs of rigid transformations containing wide rotation and translation motions to increase accuracy of the calibration.^27^ It is also shown in the literature that the error in rotation is inversely proportional to the sine value of rotation axes of either ${\mathbf {A}}$ or ${\mathbf {B}}$.^36^ Examples of wide and short motion ranges are shown in Figs. 3c and 3d, respectively.

The results are displayed in Fig. 6a. The range of magnitude in translation is incremented in steps of 3 mm from 3 to 30 mm. The magnitude of rotation range is incremented in steps of 3$^\circ$ between 3$^\circ$ and 30$^\circ$. These motion ranges are in line with the small restricted motions that are expected from robotic surgical instruments.

All cases are evaluated with six input motions, 0.5 mm in translation Gaussian noise and 1$^\circ$ in rotation Gaussian noise in both end-effector and camera motions. Motion range has a strong impact on the performance of hand–eye calibration, and this will greatly impact the results in the experiments with real data.

**Figure 7:**
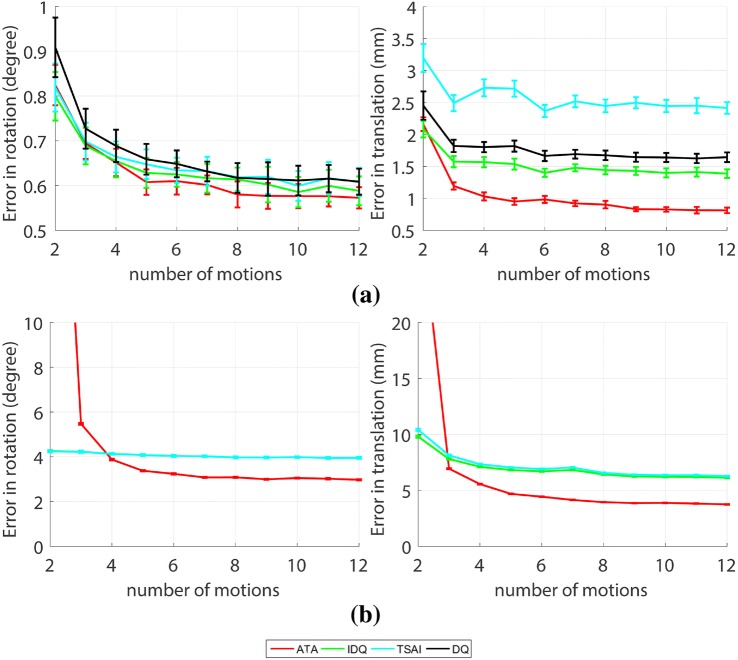
(a) Error in translation and rotation when algorithms are tested with KUKA’s data (at the number of motions of 12, $p = 2.30\times 10^{-3}$ for rotation and $p = 1.89\times 10^{-12}$ for translation). (b) Error in translation and rotation when algorithms are tested with da Vinci data (at the number of motions of 12, $p = 9.15\times 10^{-14}$ for rotation and $p = 1.18\times 10^{-16}$ for translation).

#### Increasing Number of Motions

Figure 6b shows the calibration accuracy when the number of motions is increased. As with the previous experiment, we apply a constant noise of 0.5 mm in translation and 1$^\circ$ in rotation both end-effector and camera motions. Motion range in this experiment is also kept fixed at 10 mm in translation and $10^\circ$ in rotation. It is shown in Fig. 6b that ATA outperforms the other method for any number of motions.

#### Increasing Gaussian Noise

Figures 6c, 6d, and 6e display the results when increasing zero-mean Gaussian noise is added to the camera, the robot, and stereo information. Noise in translation is increased in 0.2 millimetre steps from 0 millimetre to 2 millimetre, while noise in rotation is increased in a step of 0.2$^\circ$ from 0 to 2$^\circ$. In each simulation, 6 input motions and the same motion range as previous experiment are used.

Cameras are often considered as noise free sensors in the context of robotics. In spite of intrinsic calibration sub-pixel errors, these are normally negligible when compared to errors propagated through a robot manipulator’s kinematic chain. Robot hand pose is obtained from noisy nominal kinematic parameters that usually do not consider stress and strain in strings and cables in the presence of gravitational force. Figure 6c displays result when noise is added to end-effector’s movement. As expected, error increases with noise, however, ATA’s performance degrades at a slower rate than all the other tested algorithms.

Figure 6d shows the result when noise is added on both camera and end-effector motions. The calibration error with ATA increases at a slower rate than the other approaches for both rotation and translation.

The result presented in Fig. 6e is the closest to the scenario with real data. In the case of using stereo vision, not only the camera intrinsics and the robot kinematic parameters are noisy, but there is also an error associated with the extrinsic stereo calibration. As in the previous cases, ATA is the best performer for an increasing noise.

### Experiments with Real Data

#### Experiments with the KUKA LBR IIWA 7 R800

**Figure 8:**
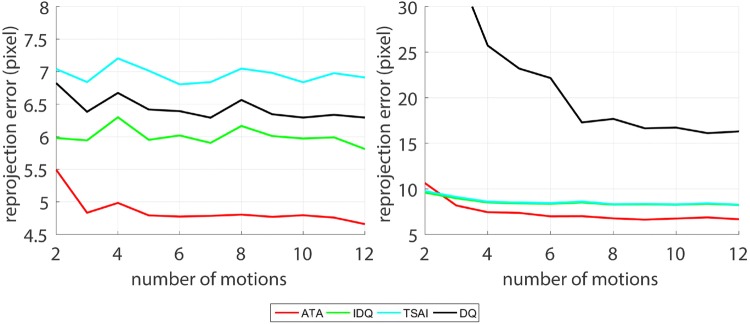
Re-projection error when predicted poses are projected back to images, KUKA data (left, at the number of motions of 12, $p = 2.01\times 10^{-18}$) and da Vinci data (right, at the number of motions of 12, $p = 3.64\times 10^{-17}$).

Figure 3c shows all camera poses that are used in the validation process. The poses are collected by moving the robot manually. We can observe that the poses are spread diversely above the grid. This kind of measurement increases the accuracy of the hand–eye calibration, according to the results shown in Fig. 6a.

As it can be observed from the results in Fig. 7a, ATA slightly outperforms existing algorithms and achieves the smallest error in camera pose estimation. The rotation error is below one degree and translation error is approximately 1 millimetre. KUKA LBR IIWA 7 R800 is well-known for its accurate positioning system and camera calibration is also known to have sub-pixel re-projection error. However, a small error in the input data can produce a significantly higher error in the hand–eye calibration, which is in accordance with the simulation results described in Fig. 6.

Figure 8 shows the re-projection error in pixels when the grid in the world coordinates is projected back to the image. The result shows that ATA has the smallest the re-projection error among all methods.

#### Experiment with da Vinci Classic

Unlike the camera pose in KUKA data, the da Vinci Classic has a rather small workspace therefore the motion range cannot be as varied as with KUKA data (Fig. 3d). The camera tends to stay in the insertion axis of the robot with limited translation and rotation movements. This makes hand–eye calibration significantly more challenging, as shown in Fig. 6a. Moreover, according to the literature, the da Vinci positioning system can only be accurate within a 5-cm cube. Therefore, the positioning system of the da Vinci is not as precise as the KUKA when the overall motion does not stay in that volume. As a result, DQ method cannot achieve stable calibration results in comparison to the other algorithms and it is then omitted in this experiment.

The calibration results and re-projection error can be observed in Figs. 7b and 8 which show that ATA also outperforms the other algorithms in this experiment. Similar to previous experiments, with da Vinci data, increasing the number of motions also increases the accuracy of hand–eye calibration, however the results converge to calibrations with higher errors than in the KUKA experiment. The insufficiently wide motion range is thus the bottleneck parameter in achieving more accurate hand–eye estimations with the da Vinci system.

## Discussion

In this work, we develop a hand–eye calibration algorithm that uses constraints derived from screw motion and stereo information to enhance the calibration accuracy in surgical robots. By implementing the constraints into the calibration, our method outperforms any state-of-the-art hand–eye algorithm in the surgical robot environment. Based on the results shown in the previous section, we believe that our developed algorithm creates an improvement in solving the calibration problem in surgical robots.

Firstly, one of the key findings of the algorithm is that our algorithm can deal with the noise in the robot parameters. The compositions of surgical robots usually cannot provide a sufficiently accurate data for the RMIS applications which require a precise positioning system. The inaccuracy in the robot parameters create a significant drift in the re-projected image as shown in Fig. 2 which in turn propagate the error to a hand-held camera motion defined in the robot coordinate frame. However, the camera motion can also be estimated using camera calibration^25^ which provides more accurate data for hand–eye problem. Therefore, one of the reasons why our algorithm works better than the other quaternion approaches is its ability to use the more accurate pose from a camera in the translation estimation instead of using the noisy orientation of the robot pose.

The second finding of the paper is the improvement of calibration accuracy as a result of using stereo constraints. RMIS usually involves using stereo camera in the procedure for navigation and localisation purposes. The algorithm makes use of the pre-calibrated stereo camera to enhance the hand–eye calibration accuracy. Our results show the clear accuracy improvements when including more motion constraints into the calibration. Furthermore, the algorithm is also tested with data from the surgical robots. This ensures that the calibration algorithm can still work well, even with the real data that contain the inaccuracy that occurs in a surgical environment.

The main impact of this finding is a robust to noise hand–eye calibration approach which offers a sub-millimetre calibration accuracy. The recovered hand–eye transformation links the information that is on the camera-end (surgical instruments, tissues and other structures at the surgical side) to the robot-end (kinematic and control). This introduces a potential of having an accurate real-time localisation of the whole surgical environment which in turn can be developed to visual servoing application, dynamic virtual fixtures and assisted instrument guidance using vision.

The only limitation of our approach is that it still cannot deal with a small range of camera motions. This problem has been well defined in hand–eye literature.^27^ It is essential to perform a wide range of camera motions in order to obtain accurate calibrations, however, in the endoscopic surgical environment and RMIS the camera motion is confined within a small volume around a pre-defined RCM. The results in Fig. 7b clearly show that the calibration accuracy deteriorates when the range of motion is smaller in comparison with the experiment with the KUKA (Figs. 3c and 3d). However, it is possible that the use of a checkerboard as the calibration object restricts the camera motion because it has to be kept in the camera frame. Therefore, using other calibration targets may increase flexibility^21^ in camera motion which will increase the performance of the calibration accordingly.

To summarise, the main contributions of this paper are an alternative solution for the hand–eye problem and a novel stereo formulation that can work with any existing approach within an iterative scheme. We provide a detailed experimental evaluation and comparison with existing approaches showing improved performance on both synthetic and real data. Our new method alternates between translation and rotation estimation until convergence using the newly developed constraints from the adjoint transformation in the hand–eye problem. It is shown experimentally that the algorithm converges regardless of its initialisation and thus it can be used as a standalone method. In the context of surgical robotics, we show that the most challenging factor in obtaining accurate calibrations is the limited workspace but despite this challenge our approach performs well. In future work, we will relax the requirements for calibration objects in order to introduce a possibility of having wider range of motions to increase the calibration accuracy and also enhance the applicability of the method to a real surgical procedure where bootstrapped approaches impede clinical workflow. For this purpose we plan to investigate other models of calibration objects, that allow to establish priors on shapes in the scene and use SfM methodologies.

## Appendix

This section shows the formula of the mapping between Lie algebra and rigid transformation. Let ${\mathbf {A}}$ be a rigid transformation with a corresponding Lie algebra ${\mathfrak {a}}$.

31 $$\begin{aligned} {\mathbf {A}}&= \begin{bmatrix} {\mathbf {R}}&\quad {\vec {t}} \\ {\vec {0}}_{1\times 3}&\quad 1 \end{bmatrix} = {{\mathrm{expm}}}{({\mathfrak {a}})} \end{aligned}$$

32 $$\begin{aligned} {\mathfrak {a}}&= \begin{bmatrix} [{\vec {\omega }}]_{\times }&\quad {\vec {v}} \\ {\vec {0}}_{1\times 3}&\quad 0 \end{bmatrix} \end{aligned}$$

Lie algebra ${\mathfrak {a}}$ is always of the form as shown in the equation above. The elements in this Lie algebra are calculated from Eqs. (33)–(36).^20^

33 $$\begin{aligned} \theta= & {} \arccos {\left( \frac{\text {trace}({\mathbf {R}}) - 1}{2}\right) } \end{aligned}$$

34 $$\begin{aligned} {[\vec{\omega }]} _{\times }= & {} \frac{\theta }{2\sin {\theta }}({\mathbf {R}} - {\mathbf {R}}^T) \end{aligned}$$

35 $$\begin{aligned} {\mathbf {V}}= & {} {\mathbf {I}}_3 + \frac{1 - \cos {\theta }}{\theta ^2}[{\vec {\omega }}]_{\times } + \frac{\theta - \sin {\theta }}{\theta ^3}[{\vec {\omega }}]^2_{\times } \end{aligned}$$

36 $$\begin{aligned} {\vec {v}}= & {} {\mathbf {V}}^{-1}{\vec {t}} \end{aligned}$$

## References

[1] Allan, M., P.-L. Chang, S. Ourselin, D. J. Hawkes, A. Sridhar, J. Kelly, and D. Stoyanov. Image based surgical instrument pose estimation with multi-class labelling and optical flow. In: Med. Image. Comput. Comput. Assist. Interv., pp. 331–338, 2015.

[2] Chen, H. H. A screw motion approach to uniqueness analysis of head-eye geometry. In: IEEE. Comput. Soc. Conf. Comput. Vis. Pattern Recognit., pp. 145–151, 1991.

[3] Chou, J. C. K. and M. Kamel. Quaternions approach to solve the kinematic equation of rotation, ${\bf {A}}_a{\bf {A}}_x = {\bf {A}}_x{\bf {A}}_b$, of a sensor-mounted robotic manipulator. In: IEEE Int. Conf. Robot. Autom., Vol. 2, pp. 656–662, 1988.

[4] Chou JCK, Kamel M (1991). Finding the position and orientation of a sensor on a robot manipulator using quaternions. Int. J. Robot. Res..

[5] Daniilidis K (1999). Hand-eye calibration using dual quaternions. Int. J. Robot. Res..

[6] Dennis JE, Gay DM, Walsh RE (1981). An adaptive nonlinear least-squares algorithm. ACM Trans. Math. Softw..

[7] Dornaika F, Horaud R (1998). Simultaneous robot-world and hand-eye calibration. IEEE Trans. Robot. Autom..

[8] Heller, J., M. Havlena, and T. Pajdla. A branch-and-bound algorithm for globally optimal hand-eye calibration. In: IEEE Comput. Soc. Conf. Comput. Vis. Pattern Recognit., pp. 1608–1615, 2012.

[9] Heller, J., M. Havlena, A. Sugimoto, and T. Pajdla. Structure-from-motion based hand-eye calibration using $l_\infty $ minimization. In: IEEE Comput. Soc. Conf. Comput. Vis. Pattern Recognit., pp. 3497–3503, 2011.

[10] Huang MZ, Masory O (1993). A simple method of accuracy enhancement for industrial manipulators. Int. J. Adv. Manuf. Tech..

[11] Intuitive Surgical Inc. ISI API User Guide: da Vinci Research Kit, 2010.

[12] Jang JH, Kim SH, Kwak YK (2001). Calibration of geometric and non-geometric errors of an industrial robot. Robotica.

[13] KUKA Laboratories GMBH. LBR iiwa 7 R800 Assembly Instructions, 2014.

[14] Li, J., G. Endo, and E. F. Fukushima. Hand-eye calibration using stereo camera through pure rotations-fitting circular arc in 3d space with joint angle constraint. In: IEEE/ASME Int. Conf. Adv/ Intell. Mechatronics, pp. 1–6, 2015.

[15] Malti A (2013). Handeye calibration with epipolar constraints: application to endoscopy. Robot. Autom. Syst..

[16] Malti, A. and J. P. Barreto. Robust hand-eye calibration for computer aided medical endoscopy. In: IEEE Int. Conf. Robot. Autom., pp. 5543–5549, 2010.

[17] Malti A, Barreto JP (2013). Handeye and radial distortion calibration for rigid endoscopes. Int. J. Med. Robot..

[18] Mourgues, F. and E. Coste-Manire. Flexible calibration of actuated stereoscopic endoscope for overlay in robot assisted surgery. In: Med. Image. Comput. Comput. Assist. Interv., Vol. 2488, pp. 25–34, 2002.

[19] Okamura AM (2009). Haptic feedback in robot-assisted minimally invasive surgery. Curr. Opin. Urol..

[20] Olinde R (1840). Des lois gometriques qui regissent les dplacements d’ un systme solide dans l’ espace, et de la variation des coordonnes provenant de ces dplacement consideres ind;pendent des causes qui peuvent les produire. J. Math. Pures Appl..

[21] Pachtrachai, K., M. Allan, V. Pawar, S. Hailes, and D. Stoyanov. Hand-eye calibration for robotic assisted minimally invasive surgery without a calibration object. In: IEEE/RSJ Int. Conf. Intell. Robot. Syst., pp. 2485–2491, 2016.

[22] Pachtrachai K, Vasconcelos F, Dwyer G, Pawar V, Hailes S, Stoyanov D (2018). Chess-calibrating the hand-eye matrix with screw constraints and synchronization. IEEE Robot. Autom. Lett..

[23] Palep JH (2009). Robotic assisted minimally invasive surgery. J. Minimal Access Surg..

[24] Park FC, Martin BJ (1994). Robot sensor calibration: solving ${\mathbf{A}}{\mathbf{X}} = {\mathbf{X}}{\mathbf{B}}$ on the euclidean group. IEEE Trans. Robot. Autom..

[25] Prince SJD (2012). Computer Vision: Models Learning and Inference.

[26] Reiter, A., P. K. Allen, and T. Zhao. Feature classification for tracking articulated surgical tools. In: Med. Image. Comput. Comput. Assist. Interv., pp. 592–600, 2012. 10.1007/978-3-642-33418-4_73 23286097

[27] Schmidt J, Niemann H (2008). Data selection for hand-eye calibration: a vector quantization approach. Int. J. Robot. Res..

[28] Schmidt, J., F. Vogt, and H. Niemann. Calibration–free hand–eye calibration: a structure–from–motion approach. In: Proc. 2005 27th DAGM Symposium, pp. 67–74, 2005.

[29] Schnemann PH (1966). A generalized solution of the orthogonal procrustes problem. Psychometrika.

[30] Selig, J. M. Lie groups and lie algebras in robotics. In: Computational Noncommutative Algebra and Applications, pp. 101–125, 2004.

[31] Shiu YC, Ahmad S (1989). Calibration of wrist-mounted robotic sensors by solving homogeneous transform equations of the form ax=xb. IEEE Trans. Robot. Autom..

[32] Spong MW, Hutchinson S, Vidyasagar M (2006). Robot Modeling and Control.

[33] Strobl, K. H. and G. Hirzinger. Optimal hand-eye calibration. In: IEEE/RSJ Int. Conf. Intell. Robot. Syst., 2006, pp. 4647–4653.

[34] Taylor CJ, Ostrowski JP (2000). Robust vision-based pose control. IEEE Int. Conf. Robot. Autom..

[35] Tsai RY, Lenz RK (1988). Real time versatile robotics hand/eye calibration using 3d machine vision. IEEE Int. Conf. Robot. Autom..

[36] Tsai RY, Lenz RK (1989). A new technique for fully autonomous and efficient 3d robotics hand/eye calibration. IEEE Trans. Robot. Autom..

[37] Zuang H, Shiu YC (1993). A noise-tolerant algorithm for robotic hand-eye calibration with or without sensor orientation measurement. IEEE Trans. Syst. Man. Cybern. Syst..

